# Epigenetic drug Gar1041 in combination with antiretroviral therapy transiently reduces the proviral DNA reservoir in SIVmac251-infected macaques

**DOI:** 10.1186/1758-2652-13-S3-O14

**Published:** 2010-11-04

**Authors:** MG Lewis, S Norelli, N Chomont, S De Fonseca, M Sgarbanti, M Collins, B Chirullo, J Yalley-Ogunro, J Greenhouse, AT Palamara, E Garaci, A Savarino

**Affiliations:** 1 1BIOQUAL, Rockville, MD 20850, USA; 2 2Istituto Superiore di Sanità, Rome, 00161 Italy; 3 3VGTI-Florida, 11350 SW Village Parkway, 3rd Floor, Port St. Lucie, FL 34987, USA; 4 4“Cenci Bolognetti” Foundation, University of Rome “La Sapienza”, Rome, 00161 Italy

## Background

It was recently hypothesized that the lentiviral reservoir in central memory (T_CM_) and transitional memory (T_TM_) CD4+ cells could be restricted by new therapies targeting pathways downstream of homeostatic proliferation or pathways associated with “stem cell–ness”, such as those developed for the treatment of leukemias. Gar1041 is one such epigenetic drug adopted in the experimental treatment of certain types of leukemia.

## Methods

SIVmac251-infected primates with viral loads stably suppressed by ART (tenofovir/emtricitabine/raltegravir) were administered for two months: Gar1041 twice daily (a starting dose of 1.5g in the first week followed by 2g in the remaining period). ART was continued during Gar1041 treatment. Proviral DNA was quantitated using a Taqman real-time PCR.

## Results

The proviral DNA content of PBMCs, which had shown no significant changes during 54 days of treatment with ART alone (p >0.05), fell below the level of detection (2 copies/10^6^ cells) in all study subjects within one month of Gar1041 treatment (p <0.05; Bonferroni’s test following significant [p=0.0003] repeated measures ANOVA). No significant changes were noticed in a control group treated with ART alone (p=0.49). The decrease in proviral DNA was associated with a significant (p=0.0156) decrease in the proportions of the T_CM_ CD4+ cell subpopulation in peripheral blood. However, both proviral DNA and the proportions of T_CM_ CD4+ rebound after two months of therapy.

## Conclusions

The present study furnishes proof of concept that pharmacological strategies may impact on the proviral DNA reservoir. However, the renewal of the phenotype T_CM_ compartment, associated with the reconstitution of proviral DNA in peripheral blood from an as yet unidentified reservoir, will require integration with other experimental approaches.

